# A Randomized Controlled Trial of the Treatment of Rotator Cuff Tears with Bone Marrow Concentrate and Platelet Products Compared to Exercise Therapy: A Midterm Analysis

**DOI:** 10.1155/2020/5962354

**Published:** 2020-01-30

**Authors:** Christopher Centeno, Zachary Fausel, Ian Stemper, Ugochi Azuike, Ehren Dodson

**Affiliations:** ^1^Centeno-Schultz Clinic, 403 Summit Blvd 201, Broomfield, Colorado 80021, USA; ^2^Regenexx, LLC, 6151 Thornton Ave #200, Des Moines, Iowa 50321, USA

## Abstract

Injectable regenerative therapies such as bone marrow concentrate (BMC) and platelet-rich plasma (PRP) may represent a safe alternative in the treatment of rotator cuff tears. This is a midterm review of a randomized, crossover trial comparing autologous BMC and platelet product injections versus exercise therapy in the treatment of partial and full-thickness supraspinatus tears. Patients enrolled into the study were between 18 and 65 years of age presenting to an outpatient orthopedic clinic with partial to full thickness, nonretracted supraspinatus tendon tears. Enrolled patients were randomized to either ultrasound-guided autologous BMC with PRP and platelet lysate (PL) percutaneous injection treatment or exercise therapy. Patients could cross over to BMC treatment after at least 3 months of exercise therapy. Patients completed the Disability of the Arm, Shoulder and Hand (DASH) scores as the primary outcome measure. Secondary outcomes included the numeric pain scale (NPS), a modified Single Assessment Numeric Evaluation (SANE), and a blinded MRI review. At this midterm review, results from 25 enrolled patients who have reached at least 12-month follow-up are presented. No serious adverse events were reported. Significant differences were seen in patient reported outcomes for the BMC treatment compared to exercise therapy at 3 and 6 months for pain, and for function and reported improvement (SANE) at 3 months (*p* < .05). Patients reported a mean 89% improvement at 24 months, with sustained functional gains and pain reduction. MRI review showed a size decrease of most tears post-BMC treatment. These findings suggest that ultrasound-guided BMC and platelet product injections are a safe and useful alternative to conservative exercise therapy of torn, nonretracted supraspinatus tendons. This trial is registered with NCT01788683.

## 1. Introduction

The rotator cuff is a structure formed by the tendinous attachments of a group of muscles that work to stabilize the glenohumeral joint. Tears of these crucial active shoulder joint stabilizers are commonly associated with trauma and age-related degeneration [1]. Rotator cuff disorders (RCD), which can be found in 30%-50% of the population aged over 50, account for over 4.5 million physician visits per year, with over 250,000 repairs performed annually in the United States [1, 2].

Traditional management of RCD includes conservative treatment which involves analgesia, anti-inflammatory drugs, corticosteroid injections, and physical therapy [3]. The rate of success with conservative treatment for rotator cuff tears varies widely from 15 to 85% [4–10]. When conservative therapy is ineffective, open or arthroscopic surgery using suture anchors in a variety of configurations is used with the goal of reducing pain and increasing function [11]. Despite traditional management with surgery to repair partial and full-thickness rotator cuff tears, several studies have shown high failure rates of tendon to bone rotator cuff repairs ranging from 30% to 94% [1, 2, 11, 12]. It can be difficult to determine which types and sizes of tears will benefit from surgical management as tear size and morphology have been shown to be poor predictors for pain and functional scores [13]. There is also evidence that surgical management does not result in superior outcomes compared to nonoperative treatment [14–16]. A recent meta-analysis of 57 randomized controlled trials found no difference between surgical outcomes compared to conservative management for treatment of full-thickness rotator cuff tears [17].

One of the difficulties with surgically repairing the rotator cuff may be on a cellular level. Animal models have demonstrated that the tendon-bone interface does not regenerate well after surgical repair with a resultant poorly formed fibro-osseous interface [18]. Research has also demonstrated that tears are prone to heal with scar tissue formation at the tendon-bone interface resulting in tissue that is weaker than the original, predisposing the rotator cuff to failure [1, 19]. As a result, practitioners have been investigating repair techniques that focus on improving the biologic healing environment of tears as well as the integrity of the tendon itself.

As an alternative treatment, early research on regenerative therapies such as platelet-rich plasma (PRP) and bone marrow concentrate (BMC) has shown promise in enhancing the healing properties of torn rotator cuff tendons, reducing retear rates over time, and improving symptoms and function [20–22]. BMC contains mesenchymal stem cells (MSCs) which are multipotent, readily available, and immune-privileged that contribute to tissue healing. When appropriately stimulated, MSCs can differentiate into bone, cartilage, fat, and tendon cells. This provides a promising mechanism to promote the healing of injured tissues [2, 23, 24]. They likely promote tendon healing through paracrine effects by secreting exosomes, growth factors, chemokines, and cytokines [25]. Several animal models have demonstrated cell-based approaches using MSCs can improve tendon repair, and when these studies are taken together, they improved histological and biomechanical properties of the tendons, indicating an increased rate of tendon healing and maturation [25, 26]. BMC and autologous-derived stem cells have also been used in the surgical management of rotator cuff repair to augment healing [22, 27–29].

In this present study, we describe a midterm analysis for a randomized controlled, crossover trial investigating a treatment for partial or complete, nonretracted rotator cuff tears using a specific protocol of BMC and platelet product injection. The primary objective of this study is to compare the patient-reported clinical outcomes for BMC versus exercise therapy.

## 2. Methods

### 2.1. Study Design

This was a prospective, randomized controlled, crossover study of symptomatic patients with chronic partial or full thickness nonretracted rotator cuff tears. Review and approval of the study protocol were obtained through the International Cellular Medicine Society IRB (OHRP Registration #IRB00002637). All patients provided informed consent prior to study enrollment.

Sample size determination was targeted at an enrollment of 50 patients with 25 in the exercise therapy group and 25 in the treatment group. This distribution was determined to have an 80% power to detect a 10-point difference in the mean change from baseline to 3-month DASH scores between treatment groups at *α* = 0.05. This estimate was based on DASH outcomes reported previously for the treatment of rotator cuff impingement with exercise therapy by Camargo et al. and the surgical treatment of rotator cuff repair along with exercise therapy by Duzgun et al. [30, 31]. Enrolled patients were randomized at a 1 : 1 ratio between the two groups. Study condition allocation was revealed by opening sequentially numbered envelopes that contained study condition based on a computer-based randomization program.

Recruitment for the study occurred throughout the local community at gyms, sports clubs/leagues, physical therapy clinics, clinic website, online research study listings, and online advertisements. Eligible patients were identified from those presenting to an outpatient orthopedic clinic with a primary complaint of shoulder pain from June 2013 to July 2017. Patients were informed of the study protocol, and those meeting the inclusion and exclusion criteria were enrolled after obtaining informed consent. Inclusion criteria included patients aged 18-65 with unremitting pain in the affected shoulder having failed conservative treatment for at least three months, significant functional disability related to symptoms, and physician exam consistent with rotator cuff tear confirmed with positive diagnostic imaging confirming partial nonretracted tear of supraspinatus tendon. Patients were excluded if they had a massive tear demonstrated by ≥grade 3 muscle strength, previous surgery to affected shoulder, prior injection-based therapies within the last 3 months, concomitant tears of multiple rotator cuff or bicep tendons, SLAP tear ≥ grade 2, type 3 acromion, significant bone spurs in subacromial space, inflammatory or autoimmune-based joint diseases, KL grade ≥ 2 glenohumeral osteoarthritis, adhesive capsulitis, symptomatic cervical spine pathology, or testing positive for malignancies. Once enrolled, patients underwent randomization into one of the two study conditions. Using a computer-generated randomization method, allocations of study condition were placed in concealed numbered envelopes and opened in order of study enrollment. Patients in the treatment group received an injection of autologous BMC and platelet products. The home exercise program was prepared by a licensed physical therapist and included stretches in all planes along with nonweighted exercises incorporating strengthening of scapular stabilizing muscles as well as the triceps and the rotator cuff muscles (Supplement 1). Upon randomization into the exercise group, patients all met with the same physical therapist to obtain the instructions for the home exercise program, including proper technique and provided a n instructional handout to take home. Compliance with the exercises and check-in was completed at 6 weeks. Exercise therapy patients completed the stretching, strengthening, and stability exercises at home, with the opportunity to cross over to the treatment group after at least 3 months of the home exercise program if desired improvement was lacking. The duration of three months of exercise therapy is in line with other published research using exercise therapy for torn rotator cuffs [4, 21, 32, 33]. Patients were followed for 24 months after receiving BMC treatment.

### 2.2. Initial Evaluation

All patients were evaluated by a physician prior to study enrollment. Coronal, axial, and sagittal MRI images of the affected shoulder were reviewed for evaluation of study criteria. Supraspinatus tears were identified in multiple planes as hyperintense signal within the supraspinatus tendon.

At initial evaluation, ultrasound examination was performed in both long and short axis to the supraspinatus tendon with a Sonosite Edge II system (SonoSite Micromaxx SonoSite, Inc. 21919 30th Drive SE Bothell, WA) and a 13-6 MHz linear phased array transducer in the modified Crass position to assess the supraspinatus. Tears were identified as hypoechoic areas within the tendon that were bursal-sided, articular-sided, or intrasubstance. Retractions were identified as a loss of tendon attachment at the expected footprint of the supraspinatus.

### 2.3. Procedure Description

Patients randomized to the treatment group, and patients after crossover, received ultrasound-guided injections of BMC and platelet products to the targeted rotator cuff tear area. All patients completed a bone marrow aspiration (BMA). The BMA procedure and processing protocols utilized for this study have been previously described [20]. In brief, bone marrow aspirate of 5-15 mL from six-nine sites was collected from the bilateral posterior superior iliac crests for a total volume of 60-90 mL into heparinized syringes. This was serially centrifuged with a resultant 1-3 mL of buffy coat collected. In addition, 60 mL of intravenous blood was drawn to isolate PRP and platelet lysate (PL). PRP was prepared by centrifugation and stored at -20°C, and PL was isolated via recentrifugation of the PRP. Total nucleated cell count (TNCC) of BMC was obtained using the TC20™ Automated Cell Counter (Bio-Rad, 2000 Alfred Nobel Dr. Hercules, CA). A 10 *μ*L sample of BMC was pipetted into 450 *μ*L of sterile water which was then mixed with 450 *μ*L of sodium chloride for a total dilution of 1 : 100. 10 *μ*L of this mixture was then placed into a microcentrifuge tube and gently mixed with 10 *μ*L of trypan blue and pipetted onto a cell counting slide. The slide was loaded into the slot of the TC20™ Automated Cell Counter which gave a total cell count per mL, as well as a live cell count per mL. This number was multiplied by the inverse of the dilution of the BMC sample (100) and then multiplied by the total volume of the BMC sample to obtain the TNCC. For the treatment procedure, under sterile conditions, ultrasound was used to localize the patient's supraspinatus tendon deficit. Under ultrasound guidance, 1-2 cc injectate consisting of 60% by volume of BMC, 20% by volume of PRP, and 20% by volume of PL was then percutaneously injected into the area of the tear.

Postprocedure, all patients were encouraged to follow a standard rehabilitation and return to activity protocol. No bracing was utilized postprocedurally. Patients were instructed to avoid any activities that caused more than a 2/10 pain throughout their rehabilitation course. Patients on days 0-3 were instructed to limit lifting/pushing and perform passive range of motion exercises 3x daily after applying heat. From 3 days postprocedure until week 4, patients were encouraged to continue 3x daily range of motion exercises with the addition of pendulum and pulley exercises and other exercises focusing on strengthening the shoulder girdle. From weeks 5 to 11, patients were encouraged to begin resistance training by starting light and progressing slowly without using resistance bands. After week 12 restrictions were lifted, patients were encouraged to perform eccentric and concentric exercises with gradual progression back to preinjury activity levels.

### 2.4. Outcome Measures

Patients completed clinical outcome questionnaires before treatment and at each follow-up visit at 1 month, 3 months, 6 months, 12 months, and 24 months. Patients completed three self-administered outcome measures in this study. The primary outcome was the Disabilities of the Arm, Shoulder and Hand (DASH), which evaluates disability, function, and symptoms for upper limb musculoskeletal conditions on a 0-100-point scale where 0 is no disability and 100 is severe disability [34, 35]. This is derived from answers to 30 questions assessing various aspects of daily and recreational activities, in addition to specific symptoms, including pain, tingling, stiffness, and weakness. Secondary outcomes included a numeric pain scale (NPS) and a modified version of the Single Assessment Numeric Evaluation (SANE). NPS directs a patient to select a whole number (ranging from 0 to 10) indicating the intensity of their pain, where 0 is no pain and 10 is the worst possible pain [36]. The modified SANE asks patients to indicate to what extent they have seen a change in their condition compared to before treatment on a scale of -100% worsened to 100% improved [37, 38]. Any scores below 0% were truncated at 0% to match the widely reported SANE scale (0%-100%). Minimal clinically important difference (MCID) was defined by a 2-point reduction in the NPS score and a 10-point reduction in the DASH score [39, 40]. Pain medications and adverse events were also recorded.

An MRI assessment of supraspinatus tears on pre- and posttreatment MRIs was performed for patients with available posttreatment imaging after at least 12 months. Pre- and posttreatment coronal and sagittal sequences of the shoulder were presented side-by-side using a computer-based randomization sequence, with physician blinded to which side represented the pre- and posttreatment MRI. Blinded to patient and image order, three grading physicians independently compared the two image sequences and identified which demonstrated the healthier tendon based on overall appearance. An additional blinded MRI assessment was also performed to measure tears by a single physician to prevent interrater variability. Tear width was measured at the widest point seen on a fluid-sensitive coronal sequence on both pre- and posttreatment MRIs. The evaluating physician was blinded to all patient data and to whether the sequences were taken before or after treatment.

### 2.5. Statistical Analysis

Linear mixed-effects models with post hoc Tukey were used to assess if outcome scores changed after BMC treatment. The effect of exercise was assessed using a Wilcoxon signed-rank test between baseline and the 1- and 3-month time points. BMC treatment was compared to exercise via Wilcoxon rank-sum tests for 1- and 3-month SANE scores, and NPS and DASH differential scores. The percent of those meeting the MCID for NPS and DASH was calculated. Linear mixed-effects models were used to determine if outcomes differed significantly between the crossover and the BMC treatment groups. MRI interrater reliability was calculated using Fleiss' kappa. All analyses were performed using R version 3.5.1, and RStudio version 1.1.456.

## 3. Results

**Figure 1:**
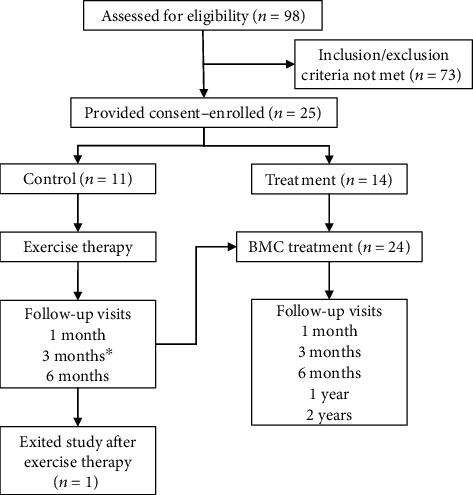
Flow diagram of study. ^∗^Exercise therapy patients were given option to cross over to BMC treatment group any time after 3 months of therapy; therefore, some 6-month outcome data was not available.

During the period of June 2013 to July 2017, 25 patients were enrolled with supraspinatus tears for this study (*N* = 14 in the BMC group; *N* = 11 in exercise therapy). The study continues to enroll new participants, but only patients who had reached at least 12 months posttreatment at the time of analysis were included in this midterm review. 24 patients have reached the 12-month follow-up time point, 17 have reached the 24-month mark, and all but 1 exercise therapy patient crossed over to the BMC treatment (Figure 1). Of the 25 total patients, 11 were diagnosed with full thickness tears of the supraspinatus and 13 were diagnosed with partial thickness tears encompassing greater than 50% of the tendon thickness. Of the partial thickness tears, 6 were considered articular-sided, 6 were at the bursal surface, and 2 were intrasubstance.

Baseline demographic characteristics were obtained revealing an average age of 47 years, average BMI of 26.4, and a male majority of 56% to 44% (Table 1). No patients in either the exercise therapy group or the treatment group have been lost to follow-up. TNCC of BMC was obtained on each study participant who received BMC treatment ranging from 271 to 2334 million, with an average of 810 million. Viability data was not recorded initially, thus data presented is for the most recent 11 study patients.

No serious adverse events were identified in any study patients during follow-up. Participants could report AEs anytime and were asked about complications at each follow-up visit. In total, there were 5 adverse events reported after receiving BMC treatment and no adverse events in the exercise group. Postprocedural pain at the shoulder and bone marrow aspiration sites were reported by two patients. One patient reported a new onset of pain and numbness in the hands and fingers starting at the 12-month time point. Two patients reported pathology in the contralateral shoulder. One patient sustained a new injury to the treated shoulder and was withdrawn from the study after 12-month posttreatment. No patient had surgical repair of their rotator cuff during the 24-month follow-up.

**Figure 2:**
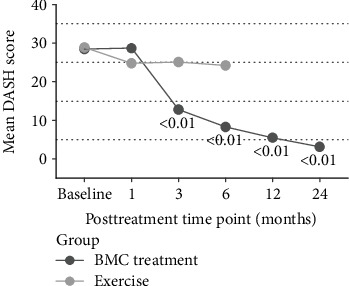
DASH scores at baseline and follow-up time points. Displayed *p* values reflect comparison to baseline. Exercise *N*: 9, 10, 5; treatment *N*: 23, 22, 24, 24, 23, 17.

**Figure 3:**
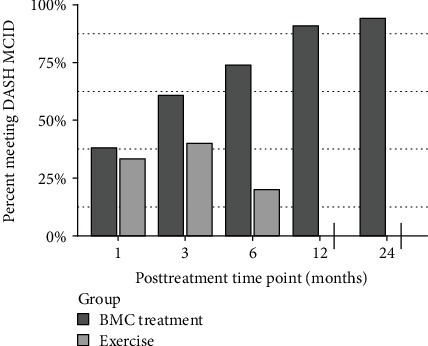
Percentage of patients who met MCID for DASH. Exercise *N*: 9, 10, 5; treatment *N*: 21, 23, 23, 22, 19, 17.

The study's primary outcome of DASH scores demonstrated statistically significant improvement after BMC treatment in comparison to the exercise therapy at 3 months (*p* < .05) and trended towards significance at 6 months (*p* = .06). DASH improvements met statistical significance compared to baseline starting at 3 months through the 24-month time point for all patients receiving BMC treatment (*p* < .01) (Figure 2). MCID for DASH was met by 61% of the BMC treatment group at the 3-month time point, improved to 91% at the 12-month time point, and was maintained through 24 months (94%). In contrast, the MCID for DASH scores in the exercise therapy group was met by 40% at the 3-month time point and 20% at 6 months (Figure 3).

**Figure 4:**
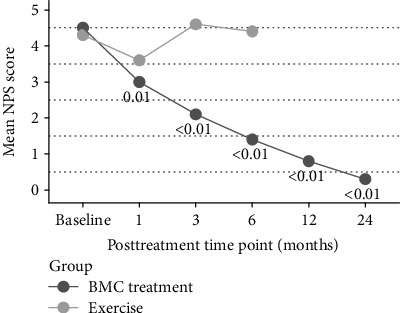
NPS scores before and after BMC treatment. Displayed *p* values reflect comparison to baseline. Exercise *N*: 9, 10, 5; BMC treatment *N*: 23, 22, 24, 23, 23, 17.

**Figure 5:**
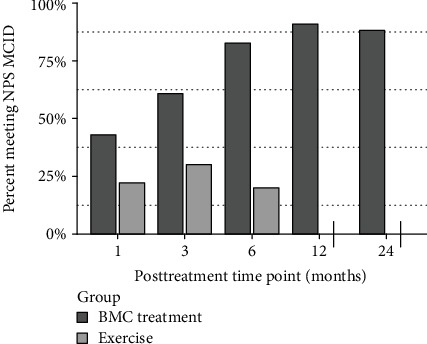
Percentage of patients who met MCID for NPS. Exercise *N*: 9, 10, 5; treatment *N*: 21, 23, 23, 22, 17.

**Figure 6:**
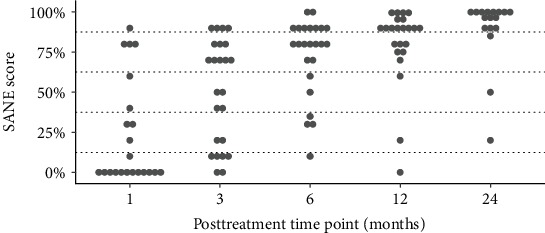
Modified SANE scores after BMC treatment at all time points.

The secondary outcome of NPS was significantly better at 3 and 6 months for the BMC group compared to the exercise therapy group (*p* < .05). NPS also reached statistically significant improvements at all posttreatment time points starting as early as 1 month (*p* < .05) in the BMC treatment group and were maintained for the remaining follow-up time points (*p* < .01) (Figure 4). Our exercise group did not meet statistically significant improvement in either NPS or DASH group before crossover (*p* < .01) compared to baseline. MCID for NPS was reached by 43% of the BMC treatment group at 1 month, which improved to 91% at 12 months and was maintained to the 24-month time point (88%). MCID for NPS in the exercise therapy group was met by 22% of patients at 1 month, improved slightly to 30% at the 3-month time point, before dropping to 20% at 6 months, for those who had not yet crossed over (Figure 5). Modified SANE scores differed significantly between groups at 3 months (*p* < .01) and exhibited significant improvements compared to 1 month in the BMC group at all later time points (*p* < .01), with a mean SANE at 12 months of 80% and 24 months of 89% (Figure 6). TNCC did not correlate to any outcome parameter at any posttreatment time point. Outcomes compared at 24 months between the crossover treatment group and the BMC treatment only group were not statistically different for DASH, NPS, or SANE (*p* > .05).

A blinded comparison of pre- and postprocedural MRIs by three physicians was performed as an additional secondary outcome. This demonstrated a consensus pick of the posttreatment MRI as the MRI demonstrating the healthier supraspinatus tendon in 14 of 15 images available for review. The interreader reliability (IRR) for this was 0.64 (*p* < .01), indicating substantial agreement [41]. The tear size assessment showed a mean decrease of 26% after BMC treatment (*p* > .05).

An additional treatment injection of autologous PRP was allowed at the physician's discretion if the patient continued to experience pain and functional impairments. Such injections were administered to 12 patients (2 patients received 2 PRP injections) at a mean of 7 months after the BMC treatment.

## 4. Discussion

This is a midterm review of data from a single-site, prospective, randomized controlled, crossover trial assessing the treatment efficacy of supraspinatus tears with BMC and platelet products in comparison to conservative exercise therapy. The BMC treatment group demonstrated significant reductions in pain and increases in function when compared to baseline and when compared to the exercise therapy group at 3 and 6 months. Those improvements continued out to the 24-month follow-up for patients receiving the BMC treatment. All but 3 patients treated with BMC reached MCID at 12 and 24 months for pain and function. Improvement in tendon pathology shown near the 3-month time point is consistent with other studies evaluating tendon healing with treatment of biologics [42, 43].

The exact mechanisms pertinent to the ability of BMC to produce these clinical results are unclear, but it is believed that MCS in the BMC provide therapeutic effects via paracrine signaling that includes immunomodulation, antiapoptosis, angiogenesis, and support progenitor cells through stimulating mitosis, proliferation, and differentiation [44]. Animal studies have demonstrated that MSC transplantations restore function and improve tissue healing [45]. Clinical studies of BMC transplantation have shown augmentation of surgical repair of the rotator cuff with subsequent decrease in retear rates [22]. Other clinical studies have revealed improvements in pain and function with simple injections in the setting of rotator cuff pathology [20, 22].

Exercise therapy in our study did not demonstrate statistically significant improvement over baseline. It should be noted that response to exercise therapy varied across patients in the exercise group, with 40% of patients meeting the MCID for DASH at 3 months, even though the overall mean does not show significant improvements. There are multiple explanations for these results. It is possible that patients were not given enough time to demonstrate clinical improvement as 10/11 patients randomized to the exercise group crossed over by the 6-month mark to the BMC treatment group, though per the study protocol all patients were required to have 3 months of conservative treatment prior to enrollment, thus extending the time of conservative therapy prior to BMC treatment. These inclusion criteria may have also led to the unintended consequence that any major improvements resulting from an exercise therapy program may have occurred prior to enrollment into the study. It is also possible that the exercise group was too small to show a statistically significant difference since this is an early review; however, significant differences were found despite the small sample in the BMC group. Finally, the patients randomized to the exercise group were given a self-guided home exercise program to be performed without the supervision of a trained practitioner which may have negatively affected compliance. The results from our home-based exercise therapy program may not be generalizable to a more rigorous physical therapy program monitored by a physiotherapist.

A comparison by three physicians was conducted for blinded pretreatment MRIs to posttreatment MRIs as a secondary outcome measure in the study. These physicians demonstrated substantial agreement that the posttreatment MRI demonstrated the healthier and less pathologic supraspinatus tendon fibers with a high interrater reliability. This finding, coupled with the tear measurements, lends evidence that there are demonstrable MRI improvements with BMC treatment to a torn supraspinatus tendon. Although the mean of tear length improvement was only 26% at a minimum of 12 months after BMC treatment, symptomatically patients were reporting feeling 80% better on average at 12 months and 89% better on average at 24 months. One possible explanation is that the BMC plus platelet product treatment decreases the concentrations of certain inflammatory cytokines, such as interleukin-8 levels in the shoulder joint fluid, which have been positively correlated to pain levels in patients with rotator cuff tears [46].

The expected natural course of a symptomatic substantial or full-thickness rotator cuff tear is to enlarge over time with an estimated 50%-82.4% of full-thickness tears and 26.1% of partial thickness tears enlarging in 12-18 months [47, 48]. There were no differences in the percentage of patients with recurrent tears after repair and patients with a tear size increase after conservative treatment, suggestive that repair does not necessarily change the natural progression of the tear [49]. Yoo et al. demonstrated no significant differences in pain and clinical outcome function scores between those with surgical repair and those who declined surgery in patients with symptomatic full thickness rotator cuff tears in which surgery was recommended [50]. The 24-month clinical outcomes after BMC injection in the current study have demonstrated encouraging data regarding improvement of pain and functional scores as well as radiographic improvement in tendon appearance.

Of the numerous components in the complex milieu of a BMC injection, the number of MSCs likely has a high impact on healing potential based on their innate healing properties. TNCC has previously been used as a proxy for the number of MSCs in an injection as it is more readily available prior to treatment than colony-forming units. While a minimum TNCC has been postulated for other areas of treatment [51], no TNCC threshold has been established in treatment of supraspinatus tears. TNCC was obtained for each BMC sample processed and varied greatly, but individual outcomes in this investigation had no correlation to the TNCC measured of each patient's BMC.

There are several potential study limitations. First, our sample size is currently small with a total of 25 subjects for this analysis. Enrollment is ongoing. We also currently have incomplete data collection at this midterm review, with a few missing data points and only 17 patients having reached the full 24-month follow-up; however, we feel it is important to present the current data since enrollment is taking longer than originally anticipated. Second, posttreatment MRI images were only available for 19 patients in the study. Three independent physician reviewers demonstrated significant interrater reliability by usually choosing the posttreatment MRI sequences as the healthier supraspinatus tendon, but the lack of complete data may limit our ability to draw conclusions on radiographic improvement. Third, a longer duration of conservative management and restricting crossover of the patients enrolled in the exercise group may have provided a more accurate comparison to conservative treatment. However, patients already had completed at least 3 months of conservative therapy prior to enrollment in the study, and almost half did not crossover until closer to 6 months after starting exercise therapy. Additionally, it has previously been showed that 3-month outcomes after an exercise rehabilitation program predicted outcomes at 2 years after commencing treatment [52]. The shorter-term conservative management and the ability to cross over was designed in part to aide subject recruitment and retention. Compliance to the home exercise program was not measured and may have been an issue, although this did not appear to be an issue noted at any follow-ups during the exercise treatment.

Fourth, both PRP and PL were combined with the BMC injection which may have influenced outcomes over a BMC injection alone. We added PRP and PL into the BMC injectate as the combination may encourage more musculoskeletal healing than any would individually [53, 54]. The growth factor, cytokine, and chemokine profiles seen in PRP, PL, and BMC are different and likely work synergistically in encouraging healing [55–58]. Multiple studies have evaluated utilizing PRP in arthroscopic repair of full-thickness supraspinatus tears without clear benefit based on clinical outcome scores but with decreased rates of failure-to-heal [59, 60]. There is only one case report on treatment of a full-thickness supraspinatus tear with percutaneous PRP injection. This demonstrated neotendon infilling and improvements in pain and function at 1 year but did not report on longer term functional or pain outcomes [61]. There is little evidence to support the use of PRP only in the percutaneous treatment of full-thickness supraspinatus tears.

Future research opportunities include directly comparing long-term exercise outcomes to rotator cuff BMC injection without a crossover group or looking at chronicity of tear prior to treatment in relationship to the outcomes of treatment with BMC. Utilization of exercise diaries would help track compliance to the home exercise program. There also needs to be research performed to see if there are different outcomes associated with different locations, types, and severities of tears.

In conclusion, according to the midterm review data from our randomized controlled, crossover trial, injections of BMC with platelet products provide significant functional gains and reductions in pain compared to a guided home exercise program in the treatment of partial and full-thickness, nonretracted supraspinatus tears. This is the first randomized controlled trial, to our knowledge, of the injection of BMC for the treatment of partial and full-thickness supraspinatus tears in a nonsurgical setting. Our findings suggest that ultrasound-guided injection of BMC and platelet product may be a safe and useful alternative to conservative management nonretracted supraspinatus tendons.

## Figures and Tables

**Table 1:** Demographic characteristics.

	Exercise		BMC treatment	
Variable	*N*	Mean ± SD/%	*N*	Mean ± SD/%
Age (years)	11	49 ± 11		46 ± 11
BMI (kg/m^2^)	11	25.9 ± 5.3		26.8 ± 3.7
TNCC (million)	10	690 ± 338	14	896 ± 517
Viability (percent)	4	92 ± 3	7	94 ± 4
Gender	11		14	
Male	5	45.5%	9	64%
Female	6	54.5%	5	36%

## Data Availability

The data used to support the findings of this study are restricted in order to protect patient privacy. Data are available from corresponding author upon request for researchers who meet the criteria for access to confidential data.

## Supplementary Materials

Supplementary Materials Instructional handouts provided to all patients in the home exercise group.
