# Supplementary figures and images for: A Randomized Controlled Trial of the Treatment of Rotator Cuff Tears with Bone Marrow Concentrate and Platelet Products Compared to Exercise Therapy: A Midterm Analysis
Source: Stem Cells Int. 2020 Jan 30;2020:5962354. doi: 10.1155/2020/5962354 (PMC7204132; doi:10.1155/2020/5962354)

HOME EXERCISE PROGRAM #1 (1-6 weeks)


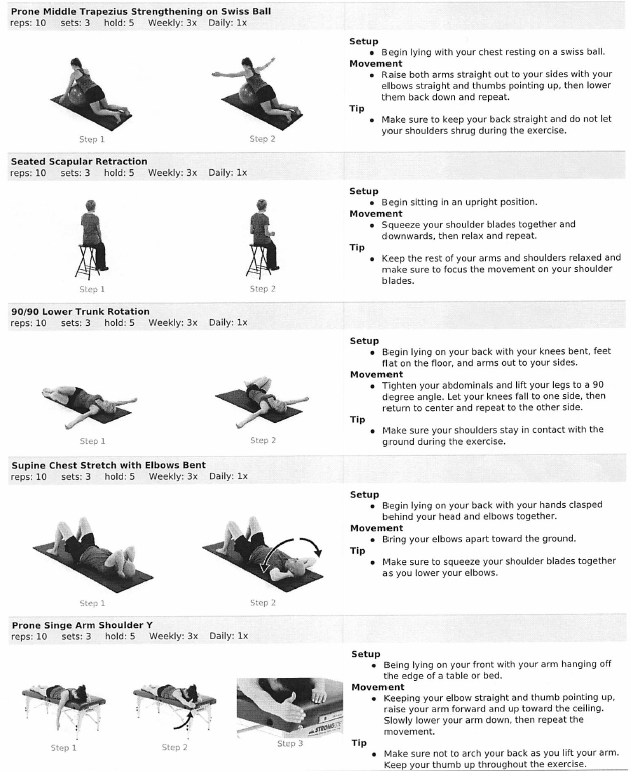


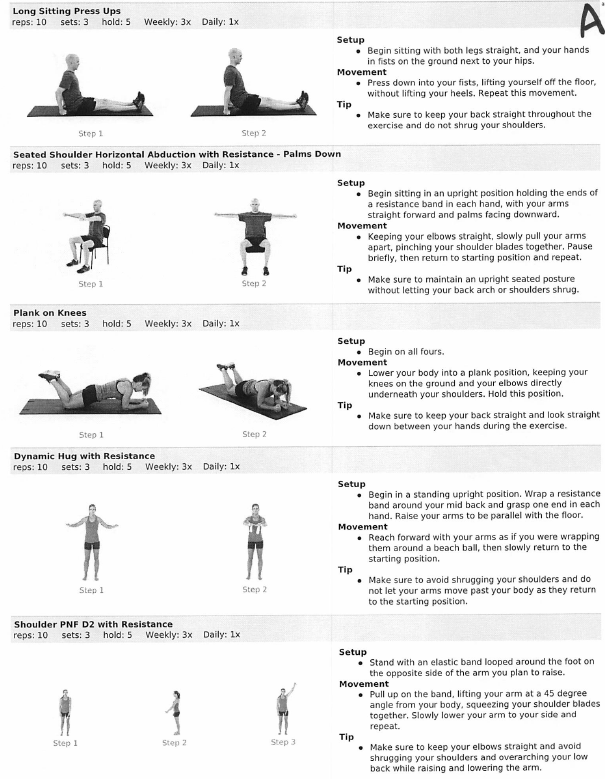


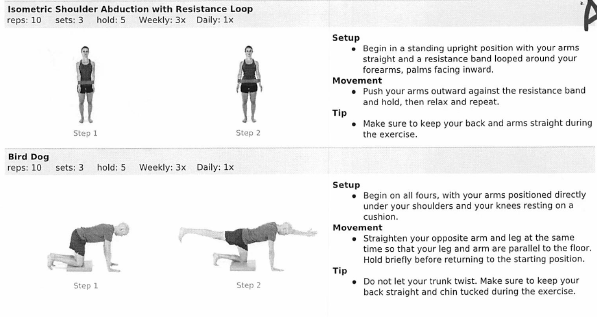


HOME EXERCISE PROGRAM #2 (7-12+ weeks)


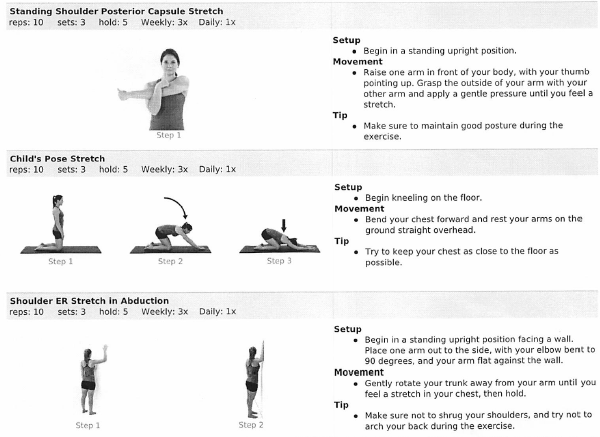


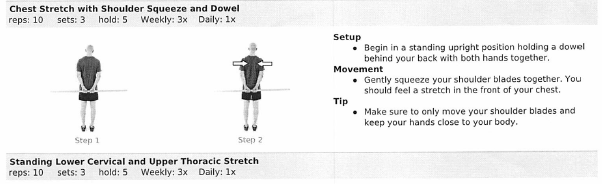


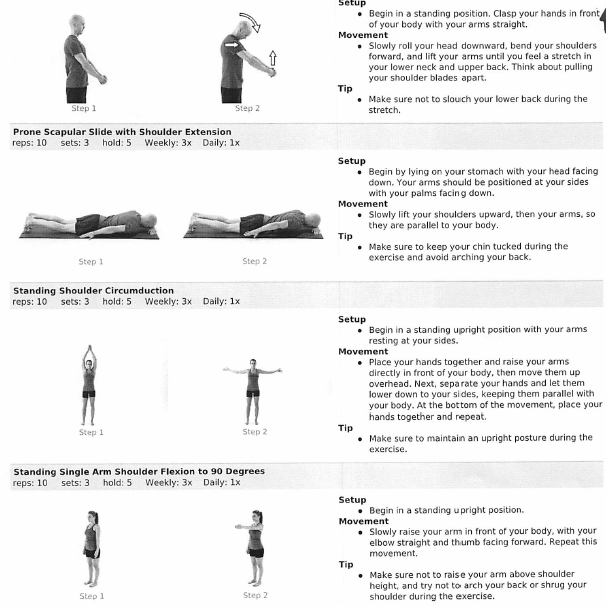


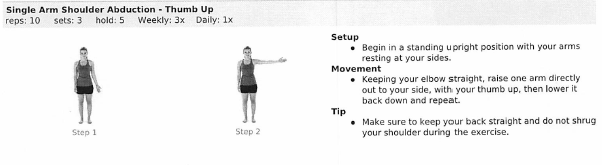


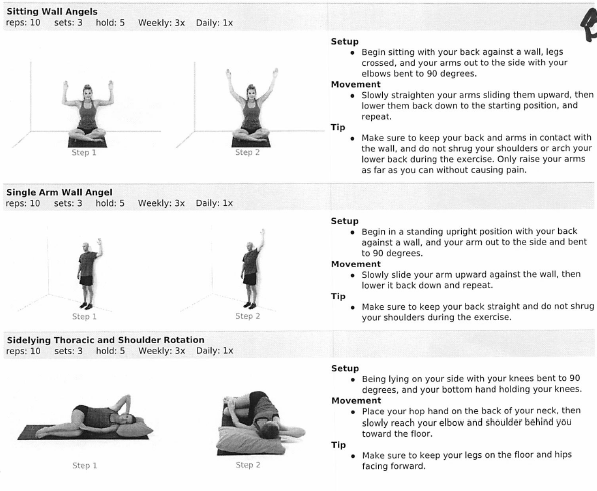

Supplement: Supplementary Materials — Instructional handouts provided to all patients in the home exercise group. [file 5962354.f1.docx]
